# Supplementary material for: Distinct neural encoding of glimpsed and masked speech in multitalker situations
Source: PLoS Biol. 2023 Jun 6;21(6):e3002128. doi: 10.1371/journal.pbio.3002128 (PMC10243639; doi:10.1371/journal.pbio.3002128)
Supplement: Attachment — Submitted filename: Response to Reviewers.pdf [file pbio.3002128.s004.pdf]

Response to Reviewers

We thank all the reviewers for their insightful and constructive feedback! We hope this submission addresses the concerns conveyed, clarifies any unclear aspects of this work, and improves the framing to better motivate this study and communicate the conclusions. In particular, we've made changes to more explicitly address the existing literature and reflect the reviewers' concerns. First, we've clarified the hypotheses generated by the glimpsing model and how we adjudicate between these hypotheses. In particular, we reference the glimpse-only and glimpse-plus-mask versions of the glimpsing model and show that our data offers clear support for the glimpse-plus-mask version, including evidence from a new analysis determining the optimal glimpse SNR threshold. Further, we've refrained from drawing conclusions beyond the extent of our data, instead detailing other possible explanations for our observations and citing literature supporting these possible explanations. In particular, we specify that our results cannot say whether any masked feature encoding occurs in a manner of top-down restoration or bottom-up repair; however, we detail how the evidence from our results points to the bottom-up repair of edges and top-down restoration of masked phonetic features. We also expand the details provided in the methods about how banded ridge was performed and what regularization values were used.

Reviewer #1

In this paper, the authors recorded neural activity in HG and STG in neurosurgical patients and tested how the neural encoding of speech features (acoustic, phonetic and lexical-borders) is affected by a) the behavioral status of the stimulus as target/non-target, and b) by the severity of momentary masking.

The sought to test neural prediction of "glimpsing" hypothesis for speech-in-noise perception (Cooke 2006 and others), according to which comprehension of masked speech (e.g. speech in noise or speech-on-speech) is afforded by the extraction of spectrotemporal features of target speech in brief periods where it is momentarily "unmasked" (i.e. the SNR changes to benefit the target speech), and these are sufficient to integrate over time and recover a fuller representation of the target speech.

This is an ambitious and thoughtful project, that provides much needed insights into the effects of local acoustics on the neural representation of speech, across multiple levels, and with fine-grained neural resolution. Results nicely show the separate roles of HG and STG in representing different levels of information and/or applying stream segregation to a mixture of voices. They also show important differences between the neural representation of 'masked' vs 'unmasked' (glimpsed) portions of the speech, which is one of the main novelties of this work.

Introduction:

> * I found the overall topic to be timely, and the study well formulated and well executed. I think the introduction could benefit from a more theoretical discussion of the proposed interaction between low-level attributes (masking) and high-level cognitive control (attention). Many important studies are cited, but it would be good to clearly state what hypotheses are generated from the glimpsing model and whether it pertains to attention or is a more low-level "default" perceptual mechanism.

Thank you for this helpful comment! We’ve changed our background of the glimpsing hypothesis and clarified the different hypotheses we hope to adjudicate between.

First, based on the description of the glimpsing model in the literature, we’ve clarified that we understand glimpsing as a low-level perceptual mechanism. Attention then operates on top of the representations derived from glimpsing. This is also indirectly supported by Cooke’s suggestion that informational masking is due to glimpsing non-target speech.

Next, this reframing has helped us identify two versions of the glimpsing model that generate separaate hypotheses that we can explicitly test with our analysis. In the glimpse-only model, only glimpses are perceived and are sufficient to support higher-order (lexical and semantic) perception. In the glimpse-plus-mask model, glimpses are perceived, but the masked portions are also interpolated to assist higher-order perception. These hypotheses not only suggest two distinct encoding patterns, but they also correspond to distinct glimpse thresholds used to compute the glimpsed and masked representations (0 and -5dB for glimpse-only and glimpse-plus-mask, respectively). Previously, we used a 0dB threshold out of simplicity and consistency with other uses of the ideal binary mask. Subsequently, we’ve reframed our analyses to adjudicate between these hypotheses by comparing the encoding patterns of these features, and we’ve included an additional analysis that measures the response prediction correlation as a function of the glimpse threshold. Our results support the glimpse-plus-mask hypothesis.

We thank the reviewer for encouraging us to be more explicit about our hypotheses, leading us to this reframing!

> * The authors often use the term "invariant representation of speech", but it's not clear exactly what they mean by this - is this an acoustic, phonetic, syntactic or semantic representation? Or all of the above? It would be useful to either define the term clearly or to choose a term that is less ambiguous.

“Invariant representation of speech” is intended to refer to a pre-linguistic representation of the sound source that contains information that is both glimpsed and masked, i.e. a representation of the sound source during multi-talker perception that is equivalent to the representation of that sound source in isolation, invariant to the degree of acoustic overlap.

We have replaced the two uses of this term. In the first, we replaced “an invariant representation” with “selective acoustic representation” to more accurately detail the conclusions of prior work. In the second, we replaced “an invariant representation of target speech” with “a complete representation of target speech, unaffected by the degree of acoustic overlap” to more accurately detail the shortcomings of prior work.

Methods

* There is a large discrepancy in the number of masked vs. glimpsed edges (18% vs. 82%). So: a) how was this accounted for in the model, and b) is this a feature of many multi-talker contexts, or did the current stimuli just happen to have many periods of non-masking? Either way, it seems important to note that most of the speech was, de-facto, unmasked.. Also, perhaps it would be nice to show a demonstration of what a masked vs. glimpsed edge looks like in the spectrogram (maybe this was intended in Fig 1A, but to me this was not sufficiently clear).

It is the case that there is a large discrepancy in the total magnitude of the glimpsed vs masked edges. This was primarily accounted for through the use of banded ridge, i.e. these features were uniquely modeled to determine the optimal regularization to maximize the amount of variance explained to account for the difference in the statistics between the various features.

We believe this balance is typical of multitalker contexts. A closer inspection of our trials indicates that the magnitude of masked edges accounted for 18% +/- 2% (mean +/- sd) of edges for both target and non-target talkers, so it appears this balance was consistent throughout our trials. Additionally, this balance is the same when computed with 10 or 100 frequency bands. Synthesizing new mixtures using speech from the same gender taker also produced roughly the same balance.

The only manipulation capable of considerably affecting this balance was the level of compression used on the spectrograms before computing the edges. We find that applying an exponent of 0.6 results in 76% glimpsed edges, an exponent of 0.3 results in 67% glimpsed edges, and logarithmic compression results in 50% glimpsed edges. For comparison, Brodbeck et al., (2020) found that approximately 67% of total edge magnitudes were assigned to glimpsed edges and 33% to masked edges; however, their spectrogram included an exponent of 0.6, and their edge detection model involved a saturation scaling factor.
Given the close agreement between our results and Brodbeck et al., (2020), we believe our use of banded ridge has sufficiently captured the neural encoding of glimpsed and masked edges.

To clarify further, it is not the case that most of the speech was completely unmasked. It is the case that most of the edges of each talker were not removed from the mixture due to the speech of the opposite talker. There are many instances in which both the glimpsed and masked edges contain energy in the same TF bin; however, the masked edges represent edge energy that is present for the talker in isolation but not in the mixture. Essentially, edge

magnitudes are mostly retained in the mixture, but quite a few edges lose some magnitude due to the mixture.

We have added a supplemental figure (Fig S1) demonstrating the extraction of glimpsed and masked edges to provide the reader with better intuition of these predictors. We hope this figure clarifies these representations.

> * The procedure for creating the glimpsed/masked phonetic spectral-temporal regressors is explained relatively clearly, but I think it, too, would benefit from a visualization of the procedure (leading to what is shown in Fig 3A). For example, it would be useful to know what words/phonemes are actually represented in the 500-ms segment shown in Fig 3A.

Thanks for this comment. We've received this feedback from others as well. We have included an example feature extraction figure (Fig 3) for glimpsed and masked phonetic features for various glimpse SNR thresholds.

> * I also wonder what the relationship between 'glimpsed edges' and 'glimpsed phoneme' is, since the phonetic features seem to be more equally distributed between 'glimpsed' and 'masked' (p.20).

This is an interesting characterization that we hadn't initially looked into. We ran an analysis that measured the proportion of the magnitude of glimpsed and masked edges in glimpsed and masked phonemes, i.e. separately for all glimpsed and masked edges, we weighed the magnitudes based on the glimpse/mask ratio of the phoneme that includes that edge and found the overall ratio of glimpsed and masked edges within glimpsed and masked phonemes.

Compared to the overall split of 82%-18% of glimpsed-masked edges, we find that glimpsed phonemes have an 86%-14% split and masked phonemes have a 76%-24% split. Masked phonemes indeed contain fewer glimpsed edges (and vice versa). These splits differ slightly from the overall split, and glimpsed edges still outweigh masked edges for masked phonemes.

However, as indicated earlier, these splits would depend on the level of compression applied to the spectrogram before computing glimpsed and masked edges. Notably, using a logarithmic compression (which results in a 50%-50% glimpsed/masked edge magnitude split) results in glimpsed phonemes with a 66%-34% split and masked phonemes with a 33%-67% split. While these different splits may influence the regularization applied to masked edges, we do not believe they would alter the results.

> * Many details are missing regarding the ridge regression procedure, including: what lambda values were ultimately chosen for each feature (in the baseline model) and were they different for each electrode or fixed per participant/all participants?

Thank you for noting the absence of these details. We have detailed each answer below and included each of these details in the manuscript.

The lambda values for each feature are as follows. Spectrogram: 42.7, glimpsed edges: 13.5, masked edges, 10.0, glimpsed phoneme onsets: 31.6, masked phoneme onsets: 17.8, glimpsed phonetic features: 144, masked phonetic features: 215, word onsets: 31.6.

Data at each electrode were normalized; therefore, the same lambda value was used for all electrodes. This is necessary to allow the direct comparison of TRF magnitudes across all electrodes, allowing simpler interpretability of the results.

In particular, these lambdas were learned in the order shown. First, the spectrogram and glimpsed edges were jointly tuned using a polar grid search of regularization values, permitting a single SVD to be computed per *theta* = arctan(lambda1/lambda2) while allowing the assessment of results with varying scales (Nunez-Elizalde et al., 2019 and example code notebook). This was done because of studies showing larger responses to edges vs spectrograms, so we wanted to jointly optimize spectrograms and glimpsed edges without bias.

The lambdas for each following feature were computed one-at-a-time, i.e. spectrogram and glimpsed edge lambdas were frozen, then masked edge models were computed sweeping over lambdas, allowing us to identify a single optimal lambda which was the frozen. Then glimpsed phoneme onsets models were computed sweeping over lambdas, etc.

> Given the very large number of features, the "baseline model" has many free parameters - what optimization procedure and/or stopping rule was used to avoid over-fitting and ensuring a single solution?.

It is the case that there are quite a few free parameters. To avoid overfitting, optimal lambdas were first selected using a 5-fold cross-validation over the entire data set. In this procedure, 4/5ths of the data was used as the training set, and the remaining 1/5th of the data was used as the test set. The use of 5-fold cross-validation for determining each lambda was intended to result in a lambda that appropriately regularizes each feature to optimally predict unseen data. For each fold, a TRF model was learned with a given regularization parameter using the SVD solution to banded ridge regression, ensuring a deterministic outcome. When all predictions had been generated with 5-fold CV, we measured the correlation between the predicted and true neural data. The final lambda was selected by determining which produced the maximum prediction correlation over all HG and STG electrodes.

Once the final lambda for a given feature was determined, the null models were computed with the frozen lambda values using 5-fold cross-validation to prevent overfitting in the null models.

> Also curious whether similar/different lambda values were selected for the same feature in different baseline models (e.g. acoustic features included in the different baseline models).

After lambdas were learned for a given feature, they were frozen when learning lambdas for later features, with the idea that this lambda results in an optimal utilization of the present information. Therefore, later features should be regularized beyond the existing optimal point, rather than by finding a new one to isolate the informational contribution of a single feature. So the same feature always used the same lambda value, regardless of the baseline model.

> And/or if the TRFs for these features, trained as part of different baseline-models, retain the same latency properties. This might be an important sanity check.

Indeed, the TRFs for the same features are nearly identical across baseline models. This was expected given that we used the same lambda value for each feature. We agree this served as a good sanity check.

Results

> * The analyses are quite elaborate and although they are well-thought out, at times the wealth of terms might be confusing for the reader. The illustration in Fig 6 is a good attempt for summarizing the main results, but the text could be improved as well. One paragraph, for example, that I found "too detailed to swallow", is the last paragraph on p. 9, which talks about "sites that only encode glimpsed phonetics or that jointly encode glimpsed and masked phonetics… STG has more diverse joint encoding properties… etc.". Many of these terms are not really defined earlier, nor are any statistics done at the single-electrode level (as far as I can tell from the methods section). So, in what sense are some sites more or less "diverse" than others? This is just one example, but I feel that much of the results section can use an additional layer of clarification.

Thank you for this important comment regarding the vocabulary used and how those terms are introduced and used.

We have changed the framing in the introduction and the results to better motivate and clarify these analyses. In the introduction, we've specified that we wish to test temporal and anatomical encoding differences to assess whether features are encoded using distinct mechanisms. In particular, temporal differences indicate additional computation needed, and anatomical differences indicate distinct neural substrates for computation and representation. In the results, we've specified that we will test differences in anatomical encoding patterns using joint selectivity and anatomical organization properties, including how we will test these differences and what they assess. We also specify how we will test these differences and how distinctions in these properties signify the use of distinct neural substrates for encoding. We hope these changes to the framing motivate and clarify the vocabulary used in our analyses.

Additionally, we apologize for not detailing the method by which single-electrode statistics were computed. We have updated the methods to specify how single electrodes were determined to be significant for a given feature. In particular, this was computed by performing a one-sample,

one-sided t-test between the prediction correlation of the baseline model and the ten feature-shuffled models.

> * Latency - the different TRFs shown for encoding different speech features show a progression of latencies (even within region), that is broadly compatible with the idea that "more complex processes occur later". For example, the peak-latency for glimpsed phonemes in HG is shorter that for masked phonemes. However, I wonder how this would play out mechanistically on a continuous basis - wouldn't it be very difficult to read-out from this early region, if the latency varied so much on a phoneme-by-phoneme basis? (especially given that the time between consecutive phonemes can be shorter than some TRF latencies..). How do the authors understand the function of the TRF peak-latency in forming "invariant" representations for speech?

This is an incredibly important question that we unfortunately cannot answer with the analyses conducted; however, we believe this line of thinking is important for future work, and we've included discussion on this topic. Gwilliams et al. (2022) show that the speech sound representation evolves over time, encoding both the phonetic feature and the time elapsed since onset. Given this evolving representation, it seems plausible that the mechanism responsible for recovering masked phonetic features could integrate the recovered representation with the correct phonetic feature and time-since-onset information. Gwilliams et al. also show that the evolution of these representations changes with phoneme duration and linguistic processing, like phoneme surprise and lexical entropy. Therefore, it seems plausible that the dynamics of phoneme sequence representation are sufficiently robust to, or potentially even leveraging, differences in phoneme encoding latency.

Regarding the function of the TRF peak latency, we interpret this as the average time at which the linear representation of feature information is maximal. The two keywords here are "average" and "maximal." TRFs are an average linear transform over all the data, so they smooth away many interesting, non-linear, stimulus-dependent transforms, and thus, we can only interpret them as such. The peak also represents the point at which the feature is most represented in the response. The feature representation may begin earlier and end later, but we characterize our results with the peak latency alone for simplicity.

> * Word-onset analysis: the "shuffled" control regressor used to compare the word-onset regressor contained "the onset of random phonemes within each word". However, does this not create an acoustic confound, since by definition these will always be in the middle of an utterance whereas words will often (although not always) be preceded by a gap in the audio? Some acoustic control might be warranted here to rule out this alternative explanation.

While this shuffling method does not create quite the same acoustic confound, we intended the presence of true (un-shuffled) acoustic information to account for this. That is to say, because the true acoustic information was included amongst the predictors (with a frozen regularization

value), any predictions that would be associated with simple acoustic cues, like a gap in the audio, would already be sufficiently accounted for.

To clarify a related point, this shuffling method allowed the possibility of the true word onset being selected as the shuffled word onset for a given word. The choice of which phoneme would be selected as the shuffled word onset was uniformly random. So the shuffled predictor was not, by definition, always in the middle of an utterance.

A previous TRF study employing word onsets used a word onset null distribution derived from random assignment amongst all phonemes (Brodbeck et al., 2018), which has temporal statistics that are less similar to the true word onsets because this method does not preserve the one-event-per-word frequency.

Given that these features are binary encoded, rather than encoded with some continuous values, we determined that temporal shuffling is necessary. We thought that time-shifting or a trial-mismatch shuffle would produce shuffled word onsets that are completely unrelated to the true stimulus and would thus show near-zero correspondence to any neural processes occuring around the once-per-word rate, other than lexical segmentation, which is what we wished to account for.

We believe our methods for controlling for acoustics and generating a null distribution align with or surpass previous methods; however, any specific suggestions beyond this are still appreciated!

Discussion

* The authors make one big "logical leap" in their inferences, suggesting that the earlier response to 'glimpsed phonetics' are a direct representation of the stimulus, whereas the 'masked phonetics/edges' are "restored" - implying some top-down "filling in" of the missing acoustics". However, this is pure speculation since no evidence is provided in the current data for the cause of this latency shift. A more parsimonious explanation could simply be delays in read-out due to perceptual load, rather than "restoration". I would encourage the authors to re-evaluate the strength of this argument, which clearly has critical theoretical implications.

Thank you for pointing out this logical leap. While there is literature to support a top-down filling-in, it is absolutely the case that our data do not present evidence for this. We have modified our framing and discussion to specify that the masked features are recovered, leaving open the possibility as to whether they are bottom-up repaired or top-down restored. We then proceed to offer interpretations of our results, as well as other literature, that point to top-down restoration rather than taking this as a conclusion of our data. We hope these changes to our framing and discussion more accurately reflect the conclusions we can draw from our data.

*   I am not sure I follow the authors logic on p. 14 that "informational masking may be due to incorrect assignment of non-target glimpses to the developing speech hypothesis of the target talker"?

We take this to mean that informational masking may arise from interference or competition in phonetic processing caused by non-target glimpses. While our results do not present direct evidence for this, they present a possible way of studying informational masking, namely by comparing the behavioral effects of informational masking with measured non-target glimpse encoding. These details have been made more explicit in the text.

*   The authors take the lack of a significant TRF to word-onsets in non-target speech as evidence for the lack of lexical processing / detection of word-boundaries. However, this conclusion does not really fit with the main message of the paper - i.e., that non-target speech is 'glimpsed' such that a detailed phonetic representation is formed, arguably providing the basis for previously reported effects of detection of words in non-target speech. This message should be clarified.

We stand by the conclusion that the lack of a significant change in prediction accuracy indicates that non-target word onsets are not *continuously* encoded. However, TRFs and prediction correlations can only produce a measure of *continuous* encoding. Nevertheless, it is possible that the *continuous* encoding of glimpsed non-target speech may support the *sporadic* encoding of non-target words, which may be sufficient to track larger structures like phrases. Further studies are needed to characterize the degree of glimpsing on non-target word or phrase perception. We hope our changes to the text are more explicit about how non-target glimpsing may support higher-order non-target speech perception through *sporadic* rather than *continuous* perception.

Reviewer #2

This paper studies neural encoding of speech in a multitalker situation. The authors refer to the glimpsing model of speech perception, whereby glimpses of individual talkers' speech in a mixture are enough bottom-up information to fill in the masked portions of speech using top-down, e.g. lexical, information. In this study, subjects are listening to two streams of speech that were mixed together. Rather than controlling the exact overlap of speech experimentally, the authors analyze the mixture post-hoc, to find glimpsed and masked portions of speech at different levels (acoustic edges, phonetic features). They then used a TRF analysis approach to see how these glimpsed and masked features are encoded in the neural responses.

The main findings are that masked features (acoustic & phonetic) are encoded later than glimpsed features, which is in line with them having to be restored top-down. Whereas masked acoustic edges are encoded/restored for both speakers, masked phonetic features are only encoded/restored for the target speaker. I think this is an interesting

finding that would deserve publication, but also a bit puzzling. There some major points that I would like to raise.

Major points:

1) Top-down or bottom-up?

If I understood the conceptual reasoning correctly, then the point of the glimpsing model is that masked speech has to be restored using top-down information. How is it that phonetic features are recovered for the target speaker only, but the acoustic edges are recovered for both speakers? In the summary figure 6, you give the example of the word 'dogs', where the acoustic evidence for the phoneme /z/ is masked. In this example the evidence for the plural marker /z/ has to come from context. If that's the case, yes, then I can recover the phoneme /z/ and the spectro-temporal edge that should have been there to cue the /z/. First of all, why would the brain even bother filling in this low-level information after the lexical access has occurred already? It seems that this would be unnecessary.

Secondly, if this were the non-target speaker and if we believe the result that the masked phonetic information (/z/) is not recovered in that case, how can the brain recover the spectro-temporal edge, which it seems to be doing? There is a discussion point in the paper that hints at a more local, low-level process to recover the acoustic edges (line 444), but I did not understand it, so some clarification would be useful. If there is a local mechanism though, to recover spectro-temporal edges, then phonetic features can in turn be constructed from that, without making use of top-down lexical information. How can we conclude from the current results that masked speech is indeed restored top-down, as you seem to be suggesting in your summary figure. Are the results more in line with some kind of local repair that enables recovery of edges for both speakers and then an attentional weighting afterwards?

Thank you for raising this point! We modified our work to specifically compare the glimpse-only model, in which glimpses alone are encoded and sufficient to support speech perception, and the glimpse-plus-mask model, in which both glimpsed and masked regions are encoded but with unique mechanisms. These hypotheses also suggest different SNR thresholds used to determine what constitutes a glimpse. While the glimpse-plus-mask model indicates the distinct encoding of glimpsed and masked speech, it makes no assumptions about how masked speech is restored. Rather, it suggests that phoneme restoration or spectral induction may be involved in this process. Subsequently, we’ve taken a more agnostic approach toward the top-down vs bottom-up nature of how masked phonetic features are encoded.

With that said, our data cannot say *how* masked phonetic features are recovered in an attentionally-selective manner or *how* masked acoustic edges are recovered in an attentionally-modulated manner–only that attention affects encoding properties in the way we

observe. Nevertheless, these distinctions in the way attention influences encoding suggest different methods for encoding these features. In particular, attentionally-modulated encoding suggests a low-level/bottom-up mechanism because such a mechanism could not strictly distinguish between target and non-target speech in a bottom-up manner. In line with previous work, we suggest that this bottom-up repair may occur through a modulation of sites preferentially tuned to the spectrotemporal properties of the target. On the other hand, attentionally-selective encoding suggests a high-level/top-down mechanism because such an attentionally-selective process would only operate on the ongoing speech hypothesis of a single target talker.

Furthermore, our added finding that the optimal SNR threshold used for defining a glimpse is -4dB indicates that there are distorted regions of the spectrogram that are still perceived as glimpses. This suggests that the restoration of masked edges may play a role in the bottom-up repair of distorted speech to enable this observed glimpsing of partially distorted speech. While the encoding of masked phonetic features, on the other hand, may play the role of recovering information that is completely overwhelmed, rather than simply distorted, by the masker.

Finally, there still exists the need to recover acoustic and/or phonetic information after lexical access has occurred. We believe this is primarily due to memory, in particular, the distinction between conceptual and perceptual memory. While lexical and semantic access may be sufficient to support a message-level understanding and produce conceptual memory of the target speech stream, perceptual memory demands different representations. In particular, we believe the representation of acoustic and/or phonetic features could be important for perceptual memory. While perceptual memory of speech is not always needed, our task required subjects to repeat the exact speech they heard, thus demanding perceptual memory. We've included the perceptual memory demands of our task as an additional limitation.

Nevertheless, the idea that masked or missing speech is restored at the level of phonemes is supported by the phoneme restoration effect. In particular, Leonard et al. (2016) showed that a phoneme replaced with noise was decodable before phoneme onset in IFG and after phoneme onset in STG. Further, the peak decoding of the noise "phoneme" occurred about 100ms later than the peak decoding of the original phoneme. The latency difference is in line with the results here and supports the notion that phonemes are still represented even after lexical access has occurred.

Overall, we do agree that our data cannot adjudicate between the bottom-up vs top-down mechanisms of masked phonetic feature encoding, and we have subsequently changed the manuscript to reflect this. Nevertheless, we still include the ideas that the observed attentional modulation of encoding and previous evidence of phoneme restoration lend themselves to the top-down mechanisms for masked phonetic feature encoding.

2) Glimpsed/masked phonetics confounded with phonetic categories?

For a given phoneme, the glimpse ratio is defined as the percentage of time-frequency bins where the energy of the talker is higher than the background. I would guess that some phoneme categories should have higher glimpse ratio because they have higher acoustic energy on average. For example vowels are usually very high in acoustic energy, followed by other sonorants (nasals, approximants); the phonemes with lowest energy are stop consonants, they are defined by a period of silence followed by a burst. This would mean that glimpsed phonetics could be highlighting vowels and masked phonetics highlight consonants. This would be an important confound, and the neural difference (latency & anatomical differences) should be interpreted differently. Namely that they reflect a difference in phoneme encoding per se (vowels vs. consonants) rather than an effect of restoration of masked phonemes.

In the paper, there is not enough information to exclude this possibility. Firstly, there's no comparison whether the glimpse ratio differs systematically for phonetic features (e.g. syllabic, consonantal). Secondly, the time courses in Fig. 3B/C represent only the average power of the TRFs to all phonetic features. We can imagine, however, that the strength of the masked and glimpsed TRFs differs systematically between different phonetic features, meaning that the average glimpsed TRF we see in 3 is more driven e.g. by vowel or sonorant features and the masked TRF by consonantal features.

Thank you for pointing out this important consideration regarding the influence of the phonetic feature identity on the degree of glimpsing. To your first point, it is certainly the case that different phonetic features have different distributions of their glimpse ratio (see below), influencing the frequency at which certain phonetic features are allocated to the glimpsed vs masked phonetic feature representations. Nevertheless, all features contains many instances that are entirely glimpsed, as well as those that are masked to different degrees.

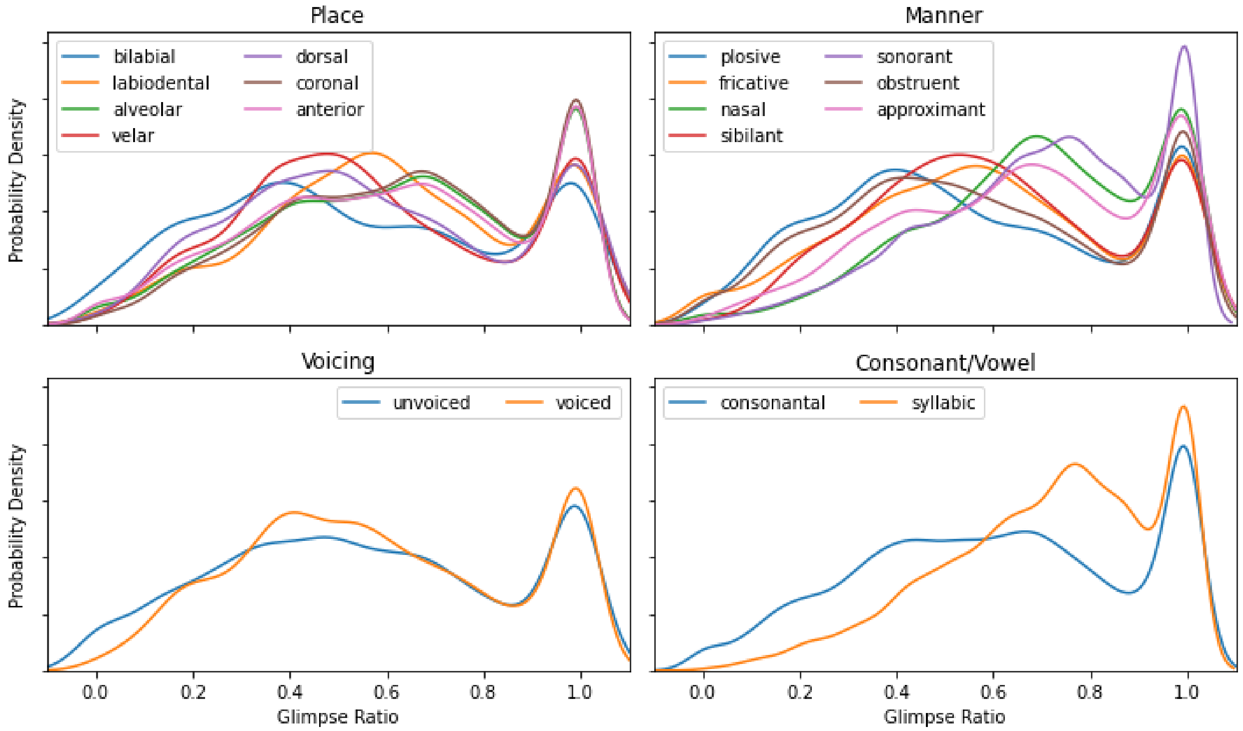

Fig. Distribution of glimpse ratio values for different phonetic features for corresponding to place (top left), manner (top right), voicing (bottom left), and consonant/vowel (bottom right) distinctions. Different features are masked more often than others. All features contain many examples that are totally glimpsed and others that are masked to varying degrees.

Even though we expect certain features (stop consonants, etc.) to be masked more often, there are still considerable periods in which these features are sufficiently glimpsed (and vice versa). Therefore, to your second point, we analyzed the individual phonetic feature TRFs to determine the latency for each feature when it is glimpsed vs masked by computing the average TRF for each feature over all electrodes in a region. This allows us to directly compare the latency for each feature when it is glimpsed vs masked in both HG and STG, revealing that this increase in latency holds across features in HG ($t=4.56$, $p<0.001$) and STG ($t=5.98$, $p<0.001$). Nevertheless, there are a handful of features that appear not to change latency or decrease in latency from glimpsed to masked. These generally represent features that do not show strong TRFs, and thus, the peak of the TRF, used to determine latency, does not differ much from the other values and is not particularly meaningful.

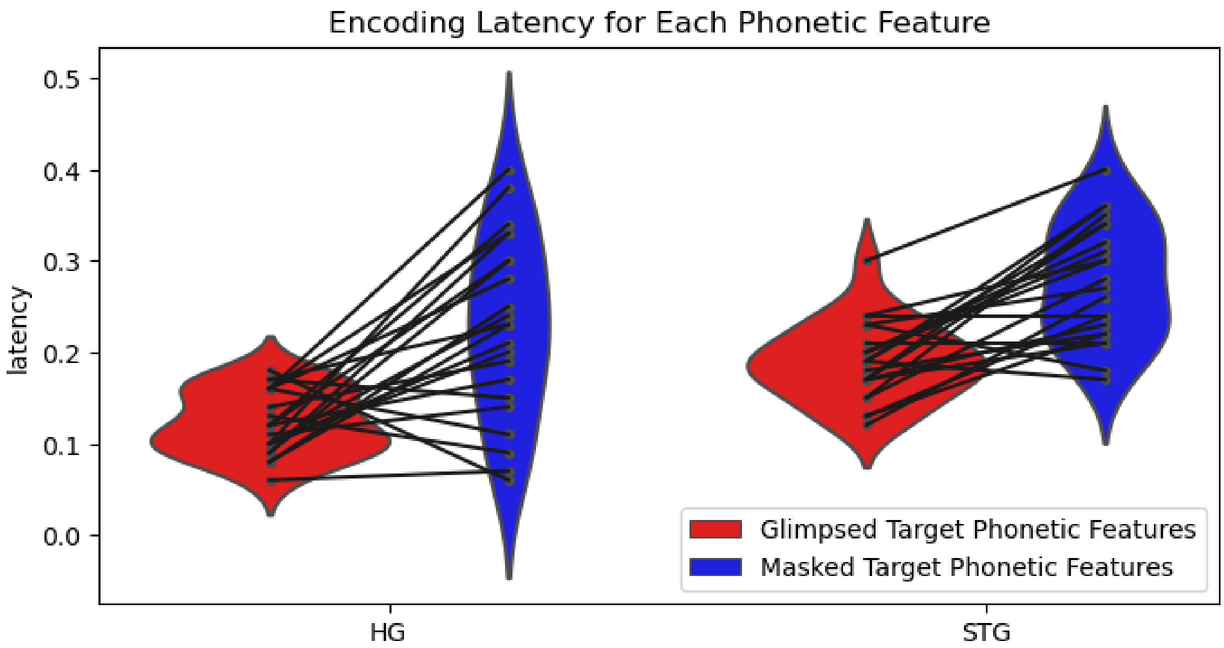

Fig. Average encoding latency for each glimpsed and masked phonetic feature for electrodes in HG and STG. Each dot/line represents a single phonetic feature averaged over all electrodes within a region-of-interest. The latency of phonetic feature encoding between glimpsed and masked features generally increases over all features within HG and STG.

To look closer, we analyzed the phonetic features that had large TRF peaks and could potentially be responsible for the difference in latency, as you suggested. Namely, we looked at sonorants, nasals, and voiced features as those likely to be glimpsed, and plosive, fricatives, and unvoiced features as those likely to be masked. On average, we find the following latencies for target glimpsed features in STG–sonorants: 200ms, nasals: 200ms, voiced consonants: 170ms, plosives: 120ms, fricatives: 130ms, and unvoiced consonants: 240ms. While voiced consonants are encoded earlier than unvoiced consonants (supporting your potential confound), sonorants and nasals come later than plosives and fricatives (against your potential confound); therefore, it is not clear from glimpse latencies alone if this confound is present.

Next, we looked at each of these features individually, comparing the glimpsed vs masked latencies for each of these target features. On average we find the following (feature: glimpse latency vs masked latency)--sonorants: 200ms vs 320ms, nasals: 200ms vs 310ms, voiced consonants: 170ms vs 340ms, plosives: 120ms vs 260ms, fricatives: 130ms vs 220ms, unvoiced consonants: 240ms vs 300ms. From this analysis, it's clear that, even at the level of individual phonetic features, glimpsed features are encoded earlier than masked features. We've added a note in our methods indicating that latency differences were confirmed to be roughly consistent for different features, rather than being driven by different features.

3) HG/STG differences robust?

The authors used a hierarchical bootstrap to control for the non-independence between the electrodes from the same subject. This is great, since it helps "confirm that the observed results were not due to responses found only in a subset of subjects or electrodes". However, they do these tests within a given electrode group, namely the HG group and the STG group. In order to interpret different results between HG and STG anatomically, in terms of a hierarchy of processing steps, we would need to make sure these differences are not due to a subset of participants contributing more to the HG or the STG group respectively, or a different electrode type (depth electrodes vs. grid).

Firstly, the paper lacks a detailed description of electrode coverage for the different subjects, so it is difficult to judge how affected the results could be by different sampling. HG is probably targeted only by depth electrodes and not by the grid, does this affect the results? Secondly, there is only one place in the manuscript where anatomic differences are explicitly tested, Fig 4. And here, the authors resort to statistics that treat electrodes as independent samples (Correlations between electrode position and encoding strength). I would encourage the authors to provide more support for claims about differential processing in HG vs. STG or anatomical gradients, considering the potential problem I mentioned.

Thank you for pointing out this missing information. We have included a brain plot showing the electrode coverage of all subjects and included information about how many HG and STG electrodes were retained from each subject.

While some participants do contribute more to the set of HG or STG electrodes, the imbalance is not particular high, nor would we expect this imbalance to influence our results. As you suggested, it is the case that all 66 of our HG electrodes were captured from depth electrodes (by necessity), while 42 of the 59 STG electrodes were captured from surface grid electrodes. Outside of clinical motivations, however, there are no reasons to believe that this would affect the results (Herff et al., 2020, Frontiers in Neuroscience; Jerbi et al., 2009, Human Brain Mapping). As far as we are aware, there are no consistent differences between the signals recorded by depth and grid electrodes, particularly because we only utilized depth electrodes located in gray matter.

Nevertheless, the points made about improving our testing of anatomical differences are well-taken. In particular, we have conducted hierarchical bootstrap statistical tests on the anatomical organization analyses in STG (Fig 5D) to account for the nonindependence of electrodes within a subject. In particular, we have applied the hierarchical bootstrap to the Spearman correlation used to assess the correlation between anatomical position and prediction correlation. As expected, this resulted in a stricter criterion for significance, but nevertheless, significance was retained for both glimpsed phonetic feature encoding vs distance from mSTG-pSTG boundary ($p=0.044$) and masked phonetic feature encoding vs PA distance ($p=0.025$).

Additional points:

- Are the regularization values (lambdas) kept the same when computing the prediction improvement for a given features. Between the full model and the model where that feature is left out?

Correct, lambdas are initially determined using the 5-fold CV and then frozen for all subsequent models. We have included more details about our banded ridge regression modeling in the methods section.

- A (supplementary) table of the phonetic features per phoneme would be very helpful.

We have provided this as a supplementary figure (Fig S2). The phonemes and phonetic features are based on the ARPABET and are an expansion of the phonetic feature set identified and utilized by Mesgarani et al. (2014).

- Line 721: sites with different response polarities. I don't understand this, since the dependent variable in the regressions is the high gamma power, which is already a rectified version of the neural signal. So what do you mean by destructive interference between sites?

Thank you for this clarifying question. We were not as precise with our language as we should have been in explaining the motivation for using TRF power (squared magnitude).

First, high gamma responses were z-scored before being used to train the banded ridge TRF models. This was done to normalize the high gamma envelope responses so that the same regularization values could be used for the TRFs of all electrodes, and thus, TRFs for separate electrodes could be fairly compared and/or averaged. Therefore, during modeling, the high gamma “envelope” neural responses contained both positive and negative values, and thus, the TRF weights reflected this, containing both positive and negative weights.

Second, we observed both facilitation and suppression of high gamma activity in response to stimulus features. In other words, we observed that some TRFs contained strongly negative weights to certain features, while most contained strongly positive weights. Nevertheless, both strong negative and positive weights indicate that the stimulus representation has a quantifiable influence on neural responses and can therefore be considered encoded in the neural response.

Therefore, the “destructive interference” included earlier is better explained by noting a) sites whose high gamma activity is facilitated by some features and suppressed by others and b) features which facilitate the activity on some sites and suppress the activity at others. We’ve clarified this in the text.

- References 60 upwards are missing

Apologies! All references are included in the revised manuscript.

Reviewer #3

This manuscript presents research aimed at examining the neural mechanisms underlying cocktail party attention. The authors record intracranial EEG from neurosurgical patients. They analyze the high gamma power of these data in different cortical regions (specifically, Heschl's gyrus and the superior temporal gyrus). In particular, the model this high gamma power activity using temporal response functions with different acoustic, phonetic, and word onset regressors. One key emphasis in the manuscript is on so-called glimpsed vs masked speech (i.e., segments of a speech stimulus that either are not or are masked by the other speech stream). They describe an interesting pattern of encoding of acoustic and phonetic speech features between masked and glimpsed speech and between target and non-target speech) - which they tie together in a nice model and very nice discussion.

Overall I found this paper simultaneously interesting and frustrating to read. My frustration mostly arises from major comment number 1 below - but also from some ambiguity and opaqueness in the methods and results sections. Meanwhile, I thought the introduction, actual results, and discussion were very interesting. Below, I list some concerns (one that I think is quite major) and queries for the authors that slightly lessened my enthusiasm over their nice discussion and model - albeit these may be answerable.

Main comments:

Comment 1

1) My main concern with the manuscript was with the glimpsed/masked dichotomy. Specifically, I had three concerns. First, I just found it very difficult to get an intuitive feel for how the two regressors are calculated and what they really look like. For example, it seems to me that one could have a strong rising edge in one speech stream with flat loud activity in the same frequencies in the second speech stream. As I understand it, this would produce a strong onset in the target and a similar onset measure in the mixture, so it would be classed as glimpsed. But, in fact, it would be heavily masked.

In the detailed example, it is the case that the target edge would be classed as glimpsed. Given that we agree that this edge would be present and audible in the acoustic mixture, we’re not clear why you would consider this edge to be heavily masked.

We do use different definitions of glimpsed and masked when it comes to edges vs phonetic features, which may be responsible for the confusion. In the detailed example, the “flat loud activity in the same frequencies” could obscure the spectral content in those frequencies. Nevertheless, increases in the amplitude of those frequencies could still be heard in the acoustic mixture, so the edge could be glimpsed while the phonetic feature could be masked.

As a contrasting example, the target could have a rising amplitude while the non-target has a falling amplitude. This would lead to a flat amplitude in the mixture, so the rising edge would be classed as masked. However, the rising amplitude in the target could have an overall amplitude larger than the falling amplitude in the non-target. Thus a phoneme here could be classed as glimpsed.

> Similarly, if both streams had rising amplitude at the same time, would they then be classed as glimpsed?

Yes, if both streams had simultaneous rising amplitudes, they would be glimpsed, as the rising amplitudes would sum additively and be clearly perceptible in the acoustic mixture.

> I don't have a good feel for this at all. I think some kind of figure and justification for the legitimacy of what you are doing here is important. I appreciate this approach has been published before, but I didn't really understand it in that paper either.

The central intuition here is that glimpsed edges are present in the mixture, while masked edges are not present in the mixture but would be present for that talker in isolation. We have included a feature extraction figure (Fig S1) to aid the explanation of glimpsed and masked acoustic edges.

Nevertheless, the motivation to include glimpsed and masked edges was a) to serve as a comparison with the MEG results of Brodbeck et al. (2020) and b) to be robust about controlling for acoustics when determining the encoding of phonetic features, both of which we believe to have accomplished.

> Second, the whole dichotomy just feels false to me in the first place. Sure, sometimes speech is completely glimpsed. And sometimes it is heavily masked. But sometimes it is in between, no? I guess this is reflected in your regressors. But dividing the data into these two clean categories just feels overly simplistic to me. (Again, though I don't have a good feel for the regressors). So I am not sure how to interpret the results in the end.

As you suggested, it is absolutely the case that speech is sometimes completely glimpsed, sometimes completely masked, and sometimes in between. This in-between-ness is indeed reflected in the regressors, as these in-between phonemes would be partially included in both the glimpsed and masked representations. While we agree it is not completely clear how these in-between-phonemes may be represented, we still believe the representative dichotomy is valuable for distinguishing these potentially separate encoding systems.

With regard to the representative dichotomy, we believe that there is sufficient prior evidence suggesting distinct phoneme encoding mechanisms, so the representative dichotomy is intended to reveal the underlying encoding dichotomy. In particular, prior evidence shows that phonemes are a) perceived categorically, even as the acoustic features differentiating phonemes are continuously modified, and b) perceptually restored, even when they are completely absent from the acoustic input.

First, experiments on the categorical perception of phonemes during speech-in-noise have shown that neural responses to exemplar vs ambiguous phonemes become more similar with large amounts of background noise (Bidelman et al., 2020). Therefore, we may expect the neural encoding of exemplar vs ambiguous phonemes to occur through the same mechanism when there is considerable noise. In our model, this would translate to the masked encoding mechanism being employed regardless of whether the phoneme was an exemplar or an ambiguous one. On the other hand, clean or minimally-noisy speech would utilize the glimpsed encoding mechanism and thus show distinct neural patterns for exemplar vs ambiguous phonemes.

As a more direct connection, behavioral evidence for the phoneme restoration effect has shown that listeners will perceive a phoneme even when that phoneme is absent from the audio. In our model, this would translate to the present phonemes utilizing the glimpsed encoding mechanism and the absent phoneme utilizing the masked encoding mechanism. This idea that present and absent phonemes have distinct encoding mechanisms has already been supported by intracranial neural evidence from Leonard et al. (2016).

With regard to the in-between phonemes, we agree it is not clear if these phonemes are represented by a single mechanism depending on which “bucket” they fall into, if these phonemes are somehow fully represented by both mechanisms, or if these phonemes are partially represented by each mechanism depending on the degree of the glimpsing or some other measure of confidence in the fidelity of the bottom-up perceived information. We found that allowing the glimpsed and masked phonetic features to be continuously valued from 0-1 (x phoneme is 20% glimpsed, thus encoded as 0.2, and y phoneme is 80% glimpsed, thus encoded as 0.8) provided better predictions than setting hard thresholds (x phoneme is 20% glimpsed, which is less than 50%, and therefore encoded as a 0, and y phoneme is 80% glimpsed, which is greater than 50%, and therefore encoded as a 1) or setting thresholds with continuous values (x phoneme is 20% glimpsed, which is less than 50%, and therefore encoded as a 0, while y phoneme is 80% glimpsed, which is greater than 50%, and therefore encoded as 0.8). This suggests a partial representation by each mechanism, but a closer analysis would be needed to make such a claim.

Further, in this revision, we have included an additional analysis testing the predictive power of glimpsed and masked phonetic features as a function of the SNR threshold used to determine what constitutes a glimpse. This revealed that using a threshold of -4dB resulted in the highest predictive power of these features. As you suggested, this potentially indicates further

distinctions in the regions of the spectrogram, namely unaffected, distorted, and overwhelmed regions (Brungart et al., 2006). Unaffected regions are what we previously referred to as glimpsed, i.e. speech that exceeds the background; distorted regions are those which exceed the glimpse threshold but do not exceed the background, i.e. the “in-between” regions; and overwhelmed regions are those which are truly masked.

In this case, separate mechanisms may be needed to repair distorted regions in a bottom-up manner vs restore overwhelmed regions in a top-down manner. This hints at the possibility that masked edge encoding may relate to the repair of distorted regions, while masked phonetic feature encoding may relate to the restoration of overwhelmed regions. The difference in the attentional modulation vs attentional selectivity of masked edges vs masked phonetic features supports this distinction; however, our results cannot say whether this is the case.

We have included a feature extraction figure to aid the explanation of glimpsed and masked phonetic features. We hope that our explanation, updated analysis, and feature extraction figures help clarify our use of these representations.

> And, third, the resolution of the spectrogram is limited (as it has to be). So segments from the two speech streams could be classed as masked based on these broad frequency bands, when, in fact, they are not masked because they are actually at different frequencies within those bands.

Thank you for pointing this out. It is the case that, after we downsampled our spectrograms (out of necessity to limit the number of regressors), we then computed the glimpse ratios using this downsampled representation. Therefore, as you mention, it is possible that segments were allocated between the glimpsed representations in a way that is not representative of a listener’s perceptual glimpsing. We have recomputed our results and re-generated our figures using glimpse ratios computed from a 100-dimensional spectrogram. This had a minimal effect of glimpse ratios overall, especially compared to the effect of changing glimpse thresholds.

Comment 2

> 2) The presentation of several of the key results was a bit ambiguous. A lot of this centered on the violin plots beside the TRFs. For example, there are some places where the non-significance of the results seems a bit implausible. Consider Fig 3B, phonetic encoding in HG for non-target speech. There seems like a clear TRF component for non-target speech here. I know the stats don't come out, but it seems likely to me that this is more an SNR issue rather than definitive evidence of absence.

Regarding Fig 3B, we’re unsure whether you’re referring to glimpsed or masked feature encoding. With respect to glimpsed feature encoding, there is indeed a clear, significant non-target TRF component, and the stats indicate that glimpsed non-target features are significantly encoded across the population of HG electrodes. With respect to masked feature encoding, there is a small TRF component; however, the population of HG electrodes does not significantly encode masked non-target features.

We rely on the change in the Fisher-transformed Pearson's correlation, as indicated in the violin plots, as the main indicator of significant encoding because these values directly tell us whether the feature-of-interest contributed to explaining more variance in the neural response. On the other hand, the TRFs tell us the approximate time course of when the feature-of-interest influenced the neural responses. Subsequently, it is possible to observe TRF components that do not contribute to an improvement in the response prediction because these components may respond to a feature due to its correlation with a lower-level feature. In particular, it is possible that we observe a TRF component in response to masked non-target phonetic features, but we do not observe an improvement in the prediction correlation due to the masked non-target phonetic features. This could be because the masked non-target phonetic features may correlate with the masked non-target acoustic edges, so including this representation as a predictor does not improve our predictions.

> I also wondered about the masked acoustic encoding in HG in Fig 2B. Is this information really being separately encoded for each speech stream? Or does the masked nature of the speech in these segments literally make it impossible methodologically to determine that they are both separately encoded. Again, some of this might relate to my confusion with respect to the regressors themselves. But it may also relate to the fact that the glimpsed/masked dichotomy is not a dichotomy at all.

The glimpsed/masked dichotomy holds stronger for acoustics, as there is a clear physical interpretation of whether or not an acoustic edge is glimpsed (present in the mixture) or masked (absent from the mixture). Nevertheless, you raise a good point about interpreting the observed encoding of masked acoustic edges. While we are unable to say whether they are truly separately encoded, we are able to say that the masked edges, which are absent from the acoustic mixture, contribute to an improved prediction of the neural responses. It is then up to interpretation as to why this might be the case. In this regard, we turn to Brodbeck et al. (2020) and agree that this recovery "could indicate that the 2 streams are completely segregated" but could also "be due to a weighting of features based on their likelihood of belonging to the attended source."

> Another example is the bottom panel of Fig 2C, where you claim that target speech is not encoded in STG (beyond the mixture). But there are significance bars for some latencies. What latencies did you use for the violin plots? Again, it seems there is signal here, but SNR might be low. I am definitely not convinced that there is enough evidence of absence to make the claim you make on lines 180-182. Same for the middle panel of Fig 2B. Indeed, this is a particularly egregious example. I'd go so far as to say that the violin plots are frankly implausible.

The correlation values shown in the violin plots were derived from predicting responses using the full TRF with lags 0-500ms. We claim that the target spectrogram is not encoded in STG based on the observation that target spectrogram encoding in this population of STG electrodes does not survive the hierarchical bootstrap. Nevertheless, there are a handful of sites that do

strongly encode the target spectrogram even though it is not encoded throughout STG. This could also be due to the specific recordings sites we've obtained. We have changed the manuscript to reflect near-significant results that indicate certain electrodes within a region may significantly encode features even though the whole region does not encode that feature.

With this said, it is unclear why you would consider the violin plots implausible for the middle panel of Fig 2B. The mixture edges significantly improve prediction correlations, and we observe TRF responses to the target and non-target edges that occur at the same time as the TRF response to the mixture edges. This suggests that only the mixture edges are encoded in HG, the target and non-target edges do not improve the response predictions, and, thus, the target and non-target TRF components we observe are caused by responses to the mixture due to the high correlation between individual talker spectrograms and the mixture spectrogram.

As mentioned earlier, the change in prediction correlation is the primary metric to assess feature encoding, especially when TRF components can be attributed to other features included in the model.

We hope this clarifies the way we evaluated the models with respect to the differences in evaluating prediction correlations and TRF components, as well as the details of the modeling process.

Comment 3

3) Incidentally, some of my initial confusion over the results was just a misunderstanding on my part - I kept seeing the horizontal significance bars for the TRFs as belonging to the TRF plot just above them - when, in fact, they belong to the TRF below. I wonder could you space the figure out a bit better to make the distinction clear so that other readers don't misunderstand things the way that I did.

Thank you for pointing out your confusion! We have adjusted the TRF plots to help clarify that the TRF significance bars align with their corresponding TRF plots.

Other comments:

1) I don't think you should be using the word "phonetics" the way you are using it - it generally refers to the study of speech sounds. I don't think you should talk about masked phonetics for example.

Thank you for this comment. We were a little careless in this use of language. We have made sure any use of “phonetics” is appropriately replaced.

2) The last sentence of the introduction is a mouthful - up to you though.

We have changed this sentence to more clearly reflect what we've tested, hopefully improving readability as well.

3) The abbreviation TRF was not defined on first usage.

We have made sure all abbreviations are defined on first usage.

4) I think you should specific "half-way rectified" in the main body of the text.

We have defined the acoustic edges as the half-wave rectified temporal derivative of the spectrogram in both the main body and the methods.

5) It is not true that reference 34 showed that responses in STG have been shown to primarily respond to amplitude change, rather than absolute amplitude. The comparison in that paper between envelope and peakEnv peakRate was blatantly unfair in terms of how the data were modeled.

We're not aware of pushback to Oganian & Chang (2019) as being flawed or misrepresentative, nor have any eLetters been submitted in response to this work to contest it. We don't feel comfortable removing this reference, as this is the first and only opposition to this work we're familiar with.

6) I don't have any great objection to the use of the word "prediction" when talking about modeling unseen neural responses…. But some people don't like it. You could consider using "we modeled high gamma" instead of we predicted high gamma. Up to you.

While we can appreciate the motivation for being selective about the use of the word "prediction" surrounding neural responses, it is standard terminology surrounding linear regression models. Nevertheless, we have changed the text to make it clear that by "prediction," we mean the modeling of unseen high gamma neural responses using stimulus features.

7) The way some of the figures are referred to is quite strange. For example, Fig 2A, bottom seems to be a reference in support of a reduction in prediction correlation. But it just refers to the spectrograms - which are mentioned much earlier in the sentence. It's unnecessarily confusing and happens a few times.

With regard to Fig 2A bottom, it is defined in the figure caption as the "example spectrograms" and in the text to refer to the spectrograms.
"First, we predicted the high-gamma envelopes from the acoustic features and measured the reduction in prediction correlation when the target, non-target, and mixture spectrograms were removed from the predictors (Fig 2A, bottom)."

In this sentence, we make no statements about the results and rather specify which features we are currently discussing. The phrase “measured the reduction in prediction correlation” specifies the method by which the values in the violin plots were computed.
To clarify that all references to Fig 2A simply indicate which feature representations are being discussed, we’ve moved the figure reference to immediately after the name of the feature, i.e. “...when the target, non-target, and mixture spectrograms (Fig 2A, bottom) were removed from the predictors.”

> 8) I don't know what "are comparable" means in the caption of Fig 2. Do you literally mean, "can be compared" - as in they are on the same axis so they can be compared. Or are you saying that they are similar?

Thank you for pointing out this ambiguity. We literally mean “able to be compared.” This is because TRF magnitudes depend on the regularization parameter, which is fixed for a given feature but not across features. Therefore, a reader can make valid comparisons of TRF magnitudes between the HG and STG TRFs but not between TRFs for different features. We’ve clarified the text to say that the TRF magnitudes use the same y-axis.

> 9) I didn't understand the sentence beginning on line 252. I don't know why no significant TRF timesteps means that encoding latency differs.

Thank you for noting your confusion here. This is a methodological consequence of the way TRF significance values were calculated. Each TRF time step is deemed significant if its distribution is significantly greater than the distribution of TRFs values for that time step in the shuffled models using the hierarchical bootstrap. If we observe a significant TRF time step, we can take that to mean that the TRF values at that time step are greater than chance, and this increase in values is consistent across electrodes. Therefore, the lack of significant TRF timesteps indicates that the increase in values is not consistent across electrodes. This indicates the encoding latency of masked phonetic features differs across HG. We’ve amended the text to clarify this.

> 10) Different latencies = different "mechanisms of encoding". Don't know what that means either - but you say it a couple of times (e.g., line 269).

Thank you for this comment. We have clarified our hypothesis more clearly specifying how we plan to evaluate whether glimpsed and masked features are encoded using the same neural mechanism. In particular, we claim that we can distinguish the encoding of different features by distinguishing the temporal and anatomical encoding patterns. The intuition here is that additional latency implies additional computation, and anatomically distinct encoding implies computations that require separate neural substrates.

> 11) "than" not "that" on line 282.

Thanks! We have changed this.

12) Not clear how you drew the conclusion that glimpsed phonetics are encoded in a feedforward manner on line 304 based on the negative correlation with distance from the boundary. Very opaque.

Thank you for this comment. This is based on the core-belt-parabelt organization of the auditory cortex, where increased distance from the primary/core auditory cortex corresponds to increased latency of processing and complexity of processing, such that bottom-up/feedforward encoding typically propagates from core to belt to parabelt areas or outward from primary auditory cortex. We hope this is now sufficiently clarified in the text.

13) The word onset stuff is very compelling/cool.

Thank you! We received broad positive feedback about this result, so we felt it was worth including to show tightly-controlled evidence for lexical segmentation in iEEG data to round out the main conclusions of this work.

14) No idea what you really mean to say by the sentence on lines 408/409.

Thanks for your comment regarding clarity here. These lines, and this paragraph, are intended to communicate that our results support the glimpsing model of speech perception in noise as a a good model for describing the neural mechanisms of speech perception in noise. We've re-written this paragraph to directly address the new framing regarding the hypotheses generated by the glimpsing model, which we hope also clarifies this sentence.

15) An extra "and" on line 458.

Thanks! We have changed this.
