# Supplementary material for: Distinct neural encoding of glimpsed and masked speech in multitalker situations
Source: PLoS Biol. 2023 Jun 6;21(6):e3002128. doi: 10.1371/journal.pbio.3002128 (PMC10243639; doi:10.1371/journal.pbio.3002128)
Supplement: Attachment — Submitted filename: ResponseToReviewers2.docx [file pbio.3002128.s005.docx]

Response to Reviewers

# Reviewer #1:

I appreciate the authors careful revision of this manuscript and their thoughtful toning-down of some of the mechanistic claims.

The addition of the parametric analysis of SNR is also useful and emphasizes the fact that masking is never binary but is always a matter of degree - and that neural encoding of non-target speech may also vary as a function of this changing degree of masking.

Another minor point that the authors may want to consider (for this paper or for future work) is how the temporal coherence across frequency bands plays in here - in the sense that if a particular speech feature (e.g. phoneme) is masked in only a portion of the spectrum, it make be easier to "recover" relative to features whose entire spectral-makeup is masked (or are more narrow band).

We’re glad our revisions were appropriate in addressing your concerns!

The temporal coherence of masking across frequency bands is a great point to bring up. It is the case that our computational definition of masking does not differentiate between masking over time vs masking over frequency. As you mention, it has been shown that glimpse duration and glimpse frequency band influences word-level speech intelligibility (Li & Loizou, 2007), possibly through the ease of recovering certain acoustic information depending on what information is present and how temporally coherent that information is. We have added this point and relevant citations to our discussion.

I also appreciate the added methodological details regarding model optimization. Since there are many choices and options for conducting these analyses, full transparency here is critical.

We absolutely agree, and appreciate your request that we provide these details at a fine scale. We’ve certainly been frustrated with incomplete modeling details in other papers!

The discussion is now more cautious and well balanced and even though many open questions remain, I believe that the manuscript in its current form makes a useful contribution to the growing literature attempting to 'reverse-engineer' how the brain encodes and processes concurrent speech.

Thank you for all of your helpful feedback that was essential for us to reach this point!

# Reviewer #2:

The authors addressed my specific concerns well.

I'm happy to see that the glimpsed vs. masked difference mostly generalizes across the different phonetic categories (major point 2): this would have been a major concern for me.

It's good to see that almost all subjects contribute both to the HG and the STG groups of electrodes (major point 3) and that the analyses on anatomic gradients seem to hold taking subject dependencies into account.

We’re glad our revisions and responses helped ease these specific concerns, and thank you again for raising them! Our work is better off for it.

Major point 1: I think the new introduction is much clearer on the theoretical scope of the current work, namely the difference between a glimpse-only and glimpse-plus-mask model. I have only a comment on the accessibility of the concepts:

The notion of glimpsed and masked edges is central to this work, but it's hard to understand when it goes into the details. After a few month since reading the first version of the manuscript, it took me quite some time to understand the different features. Especially in Fig. 2A it was very confusing to have 'glimpsed edges' for the mixture. I had to go back and forth between the methods, the main text and the figure to figure out how the features relate to each other. I realized only after a while that there is a new Supp Fig 1, with illustrations that explain this very well. In my mind, this belongs in the main text. Together with the illustrative examples in the new figure 3 (which illustrates the glimpse ratio for phonetic features), this would make the paper self-contained and accessible. That's a suggestion of course.

Thank you for noting your confusion here. Glimpsed and masked edges are indeed central for robustly modeling acoustics to show that the observed phonetic feature encoding is not due to acoustics (Daube et al., 2019), as well as for showing the encoding of glimpsed and masked features that follow different patterns of attentional modulation. We can appreciate your confusion with “glimpsed” mixture edges, as glimpsed edges are defined based on whether or not they are present in the mixture, so all mixture edges are glimpsed by definition. We have clarified in the text that “all mixture edges are considered glimpsed.”

The glimpsed and masked edge results also offer a direct comparison with Brodbeck et al. (2020). In fact, our former Supp Fig 1 demonstrates a very similar example to Figure 4A in Brodbeck et al. (2020), which is why we included it as a supplement, rather than replicating a panel from another paper. However, we appreciate the inclination to make this work self-contained and have included the former Supp Fig 1 as Fig 2A to make the details of these features more immediately obvious to the reader.

The new analyses on the glimpse threshold are a cool idea. As the authors mention, it suggests interesting differences between clear preserved edges, distorted edges and fully masked edges. In terms of the neural data, this is very exploratory, and should be clear from the paper. At the very least, there should be some tick-labels showing the magnitude of this effect: I assume it is small, and not statistically different (between -4dB and the original 0dB). That doesn't mean it's not meaningful, just the experiment wasn't designed to specifically test this idea. But there should be some way for the reader to compare the magnitude of this effect to the other reported model comparisons.

We’re glad you appreciate this added analysis! We acknowledge that this analysis is “exploratory” in that it is a post hoc analysis of what constitutes a glimpse based on a dataset collected with a very simple experimental paradigm. We have changed our text to reflect that this was a post hoc analysis and not part of the experiment itself. In particular, we changed the text, “First, we sought to determine how much speech could be distorted for listeners to remain able to perceive that speech as glimpsed” to “First, we sought to determine how much speech could be distorted while still being optimally classified with clean speech.”

Additionally, we changed Fig 3C to include the prediction correlation values on the y-axis as well as horizontal bars indicating statistical significance between the prediction correlations from different SNRs, as we did observe significant differences.

The supplementary video didn't work for me.

We’re sorry to hear that. We went back to the PLoS Editorial Manager, re-downloaded the supplementary video, and confirmed it played back as expected using both VLC Media Player and QuickTime Player. Nevertheless, we re-uploaded the video in case that may help.

# Reviewer #3:

Many thanks to the authors for their efforts in responding to my previous comments. Their clarifications where helpful in many places. However, I still have some residual unease with the way in which glimpsed and masked speech has been defined. I feel that the authors and I have been speaking past each other a little on this issue, so I will have one more try at explaining my concern here.

The authors are defining glimpsed and masked speech based on edges. I understand that much. But that approach just seems deeply flawed to me.

We apologize for giving this impression. We have not defined glimpsed and masked speech based on edges, rather we have separately defined a) glimpsed and masked edges and b) glimpsed and masked phonetic features. In one feature set, we define glimpse and masked edges that are based on the positive values of the change in amplitude (derivative) of the spectrogram, and these edges are classed as glimpsed or masked depending on whether those increases in amplitude are present or absent, respectively, in the acoustic mixture. In another feature set, we define glimpsed and masked phonetic features that are based on the distinctive features of phonemes and are quantified as glimpsed or masked depending on how much of the spectrogram of that talker’s phoneme exceeds that spectrogram of the background. Note that edges are not used in the quantification of glimpsed and masked phonetic features.

We mentioned in our first response that “we do use different definitions of glimpsed and masked when it comes to edges vs phonetic features, which may be responsible for the confusion.” To clarify this further, we use the same vocabulary (glimpsed, masked) to refer to these separate representations (edges, phonetic features) since they convey the same intuitive meaning, i.e. glimpsed regions are easily perceptible by the listener in the mixture, while masked regions may not be perceptible by the listener in the mixture, even though these terms utilize separate computational definitions for each separate feature.

With respect to edges, the glimpsed/masked distinction is relatively intuitive: the glimpsed edges of a talker are those which would be present for the talker in isolation and are also present in the acoustic mixture, while the masked edges of a talker are those which would be present for the talker in isolation but are absent in the acoustic mixture. We included S1 Fig (moved to Fig 2A) to visually demonstrate this distinction.

With respect to phonetic features, the glimpsed/masked distinction is less intuitive to define. In particular, the glimpse ratio of a phoneme is the ratio of time-frequency bins of the spectrogram of that phoneme which exceed the background by the SNR threshold. This is why we introduce the SNR threshold: to determine by how much the time-frequency bins of the talker’s spectrogram must exceed the background in order for those bins to be considered “easily perceptible by the listener in the mixture” (the “intuitive” meaning of glimpsed provided above).

We hope these descriptions and our explanations of the examples below clarify our use of glimpsed and masked representations.

I'll revisit two examples from my previous review. The first was my previous point number 1. I will try to illustrate that point again with a silly extreme example. Imagine the masker speaker is screaming a steady perfect 1kHz tone in my ear at 110dB. Because their "speech" in that moment is flat, there is no edge. Meanwhile, the target speaker gently increases the energy in their speech from, say, 60 to 65 dB at 1kHz at that time. So there is an edge. And there will be an edge in the mixture. As such, according to the authors, this would qualify as glimpsed speech. However, given the masker is screaming in my ear, I don't see how this can possibly be construed as glimpsed speech. Can the authors please explain to me again what I am misunderstanding? The response to the previous review just says that the authors are "not clear why [I] would consider this edge to be heavily masked." How can it meaningfully be considered glimpsed if someone is screaming in my ear at 110dB?

It is essential here to distinguish between glimpsed edges and glimpsed phonetic features. Just as you say, the target gently increasing the energy in their speech would lead to a glimpsed edge, which we believe would be perceptible, as the auditory cortex appears to respond to changes in energy rather than the total amount of energy (Oganian & Chang, 2019; Khalighinejad et al., 2019 *Nat Comm*). However, if we pretend that these 1kHz tones represent phonemes, it is clear that all frequency bins of the target “phoneme” do not exceed the glimpse threshold, and thus would be considered masked. So per your example, the target producing a 1kHz tone that increases from 60dB to 65dB while a 1kHz tone at 110dB occurs in the background would produce a glimpsed target edge but would lead to a target phoneme glimpse ratio of 0 and mask ratio of 1, i.e. that “phoneme” is considered completely masked.

Furthermore, the method the authors are using seems to run counter to what the authors say in the abstract - specifically they say that glimpses "are spectrotemporal regions where a talker has more energy than the background". But that is not how it is being operationalized in the analysis. Can the authors please help me understand this discrepancy?

Our abstract text is accurate; we operationalize glimpses as spectrotemporal regions where a talker has more energy than the background through the computation of the glimpse ratio of each phoneme. We included Fig 3 to visually demonstrate this computation.

In the second example we previously discussed, the authors say that if the two streams had rising amplitudes at the same time, then both streams would be glimpsed. Again, I don't understand. If they are both rising at the same time, then they are - almost by definition - both masking each other, no? To take an extreme example again - if I was listening to two speakers saying different things, but with highly similar timing - then, according to the operationalization in the study, both of these streams would count as glimpsed? But they would clearly both be masking each other heavily.

Again, it is important to distinguish glimpsed edges from glimpsed phonetic features. As we mentioned previously, if the two streams had rising amplitudes at the same time, the edges of both talkers would be glimpsed because those increases in amplitude would sum additively and be audible in the acoustic mixture.

The degree to which the phonemes of each talker in your example would be considered glimpsed then depends on the specific frequency overlap of each of the phonemes and the relative volume of each talker. If, for example, both talkers are speaking at the same average volume and one talker is making an /f/ sound while the other is making a /sh/ sound, the /sh/ sound could be glimpsed while the /f/ sound could be masked since these sounds occupy the same frequency band and we would expect that /sh/ sound to contain more energy in those frequency bands than the /f/ sound.

Here, when we say that “the /sh/ sound could be glimpsed…” that is operationalized as the phonetic features of the /sh/ sound being encoded in the glimpsed phonetic feature representation using a large (close to 1) glimpse ratio and encoded in the masked phonetic feature representation using a small (close to 0) mask ratio.

To a good extent, this point hits on the exact purpose of this work. When separate talkers are both speaking with highly similar timing, the degree to which each talker masks the other depends on the frequency content of each talker at each moment in time. When they differ in frequency content, it is possible to clearly perceive (“glimpse”) both talkers; however, if they overlap in frequency content, one may mask the other depending on their relative volume. The difference in the neural encoding of the phonetic features in each of these situations is exactly what we sought to study.

Of course, as mentioned in the previous response, this co-occurring speech is not always clearly glimpsed or masked but somewhere in-between, which we reflect in the continuous nature of the glimpse/mask ratio which is reflected in our regressors.

Again, I am sorry to say, I remain uncomfortable with the definition of glimpses and masks that is central to the whole manuscript. Neither the authors, nor the other reviewers seem unhappy with it though. So I don't want to stand in the way of publication necessarily. But I do think it is worth considering some further explication and justification of the approach here. I study these kinds of questions quite a lot. And I feel like if I am this uncomfortable with the whole thing, then others will be too. Again, maybe it is just me. If the authors think that is likely to be the case, then, again, I won't stand in the way of publication.

We hope the explicit detailing of the distinction between glimpsed/masked edges vs phonetic features helps clarify both the intuitive and computational definitions of “glimpsed” and “masked” we’ve used here. To make it more explicit that the method by which we compute glimpsed and masked phonetic feature representations is distinct from the method by which we compute glimpsed and masked edges, we have changed the text, “measuring the proportion of time-frequency components of a phoneme greater than a given threshold” to “measuring the proportion of time-frequency components of the phoneme’s spectrogram greater than the background spectrogram by a given threshold.”

I still think there is a disconnect between the TRF plots and the violin plots in several of the figures. Several figures show clear deviations from zero in the TRF plots (at a narrow range of latencies). But, because the authors have chosen to assess prediction accuracy based on a broad range (0-500 ms) of latencies, the overall effect (as depicted in the violin plots) does not come out as significant. There is clearly some relevant activity in some of these cases though - according to the TRFs. Reporting no effect based on the violin plots (which are based on *too* broad a latency window) just feels like embracing the null to me.

The data depicted in the violin plots are computed from the change in the Fisher-transformed Pearson’s correlation of the electrodes in a given region when that feature is removed from the set of predictors used to predict the neural response. When overall effects do not come out as significant, this indicates that the subjects and electrodes analyzed in a given region do not collectively experience a drop in prediction correlation as a result of removing that feature from the set of predictors. Nevertheless, it is the case that some electrodes do lose prediction accuracy when removing a certain feature, even though all of the electrodes in a region do not do so collectively, and some of the effects that you pointed out were found to be significant before conducting the hierarchical bootstrap.

Additionally, the acoustic features used here are highly correlated with each other. In multiple regression, this is known as multicollinearity. When training TRFs to predict neural responses from multiple correlated features, we can expect the overall predictive power of the model to accurately reflect how well these features predict the neural response; however, we cannot expect the TRF values themselves to tell us how predictive a specific feature is. So when an unencoded feature is correlated with an encoded feature, we would expect the TRF of the unencoded feature to resemble the TRF of the encoded feature.

As a simple example of this, imagine a “full stimulus” impulse train with impulses occurring every 1 second, and a “neural response” that contains identical evoked responses to each of the impulses. We could predict this “neural response” perfectly from the “full stimulus,” and the TRF would look like the evoked response. Now imagine a “partial stimulus” impulse train with impulses occurring every 2 seconds. We cannot predict the neural response any better by adding the “partial stimulus” to the “full stimulus” in the set of predictors; however, training a regression model to predict the “neural response” from both the “partial stimulus” and “full stimulus” would lead to large TRFs for both the “partial stimulus” and the “full stimulus,” as there are clear time-locked responses to each of these stimulus features. On the other hand, if the “neural response” contained identical evoked responses that were doubled in amplitude every 2 seconds, both the “full stimulus” and the “partial stimulus” would be needed to form the most accurate prediction of the neural response, and both stimulus features would have a large TRF.

We hope that this toy example clarifies why we must first rely on predictive power to assess whether stimulus features are encoded, only after which can we interpret the weights of the TRF.

Therefore, the instances in which the TRFs deviate from the baseline but we do not observe a significant change in prediction correlation across the region could be caused by some combination of two main reasons.

1. The TRF values are driven by a small number of electrodes, or electrodes in only a subset of subjects, that do respond to the feature in question; however, not enough sites in the region respond to the feature in question for the overall encoding to be significant. This explanation was offered in our revision (“some enhancement of the target spectrogram may occur at specific sites but not throughout the region”).

We have expanded this explanation in the text (“However, target spectrogram encoding is approaching significance, and some sites show a change in prediction correlation comparable to the mixture spectrogram, indicating some enhancement of the target spectrogram may occur at specific sites but not throughout the region”).

2. The TRF values for the feature in question are driven by a correlation with another feature that does show robust encoding, while the feature in question does not show robust encoding. This explanation was also offered in our revision (“While the individual talker TRFs contain significant sections, they align closely with the mixture TRF, suggesting they capture the same information”).

It is not likely that training these models with too broad a range of latencies hurts some features more than others, some talkers more than others, or some electrodes more than others. Thus, observing some clear effects while claiming less clear effects should be considered more closely is inconsistent.

We recognize that some null results may seem odd, e.g. lack of significant target spectrogram encoding in STG. However, we sought to characterize whether the target spectrogram is encoded *throughout* our population of electrodes in STG, and it only appeared in a subset of electrodes that was also limited to a subset of subjects, leading to a non-significant hierarchical bootstrap result. This result is also consistent with previous literature showing late acoustic encoding is more driven by edges than spectrograms (Brodbeck et al., 2018; Brodbeck et al., 2020).

Given the reasons we may expect to observe TRFs for features that are not robustly encoded, we would rather be more careful about overinterpreting our results, rather than liberally claim effects. This is primarily why we utilized the hierarchical bootstrap: to confirm that our results are reliable across subjects and electrodes, and we are careful not to point to false positives driven by a subset of electrodes.
